# Regulation of Kinetic Properties of Chemical Hydrogen Absorption and Desorption by Cubic K_2_MoO_4_ on Magnesium Hydride

**DOI:** 10.3390/nano12142468

**Published:** 2022-07-19

**Authors:** Xinglin Yang, Jiaqi Zhang, Quanhui Hou, Xintao Guo

**Affiliations:** 1 School of Energy and Power, Jiangsu University of Science and Technology, Zhenjiang 212003, China; zhangjiaqi7102@163.com (J.Z.); gxt960308@163.com (X.G.); 2 School of Automotive Engineering, Yancheng Institute of Technology, Yancheng 224051, China; hqhdyx66@ycit.edu.cn

**Keywords:** MgH_2_, hydrogen storage materials, K_2_MoO_4_, kinetic properties, reversibility

## Abstract

Transition metal catalysts are particularly effective in improving the kinetics of the reversible hydrogen storage reaction for light metal hydrides. Herein, K_2_MoO_4_ microrods were prepared using a simple evaporative crystallization method, and it was confirmed that the kinetic properties of magnesium hydride could be adjusted by doping cubic K_2_MoO_4_ into MgH_2_. Its unique cubic structure forms new species in the process of hydrogen absorption and desorption, which shows excellent catalytic activity in the process of hydrogen storage in MgH_2_. The dissociation and adsorption time of hydrogen is related to the amount of K_2_MoO_4_. Generally speaking, the more K_2_MoO_4_, the faster the kinetic performance and the shorter the time used. According to the experimental results, the initial dehydrogenation temperature of MgH_2_ + 10 wt% K_2_MoO_4_ composite is 250 °C, which is about 110 °C lower than that of As-received MgH_2_. At 320 °C, almost all dehydrogenation was completed within 11 min. In the temperature rise hydrogen absorption test, the composite system can start to absorb hydrogen at about 70 °C. At 200 °C and 3 MPa hydrogen pressure, 5.5 wt% H_2_ can be absorbed within 20 min. In addition, the activation energy of hydrogen absorption and dehydrogenation of the composite system decreased by 14.8 kJ/mol and 26.54 kJ/mol, respectively, compared to pure MgH_2_. In the cycle-stability test of the composite system, the hydrogen storage capacity of MgH_2_ can still reach more than 92% after the end of the 10th cycle, and the hydrogen storage capacity only decreases by about 0.49 wt%. The synergistic effect among the new species MgO, MgMo_2_O_7,_ and KH generated in situ during the reaction may help to enhance the absorption and dissociation of H_2_ on the Mg/MgH_2_ surface and improve the kinetics of MgH_2_ for absorption and dehydrogenation.

## 1. Introduction

With the massive utilization of fossil energy and the increasing environmental pollution, traditional transportation methods such as ships and automobiles have faced unprecedented challenges. With the implementation of the zero-carbon emission goal, the status of hydrogen energy in many energy sources has been further improved. Unfortunately, the low density, flammable, and explosive nature of hydrogen energy make its storage very difficult. Therefore, the key to the successful realization of the “hydrogen economy” is the development of efficient hydrogen storage materials and corresponding methods [1]. The ideal hydrogen storage material should be able to carry out reversible hydrogen de/absorption at mild temperatures and have a high hydrogen storage capacity. Among many solid hydrogen storage materials, magnesium hydride (MgH_2_) stands out because of its high volume and mass hydrogen storage capacity (110 g/L, 7.6 wt%) and good reversibility. In addition, the low price and simple preparation method of MgH_2_ are also one of its advantages that cannot be ignored. However, the slow kinetic properties and stable thermodynamic properties of MgH_2_ seriously affect its adsorption and desorption performance. In order to solve this technical difficulty, the current mainstream methods can be classified into four kinds. They are catalyst doping, construction of the composite system, nanocrystallization, and alloying of hydrogen storage materials [2,3,4,5,6].

It is gratifying that catalyst doping, as the most simple and effective means at present, has been widely proved to regulate the kinetic performance of MgH_2_ and reduce its chemical energy barrier to a certain extent, to realize the rapid absorption and desorption of MgH_2_ at lower temperatures. Ni-based [7,8,9,10], Ti-based [11,12,13], Fe-based [14,15,16], Mn-based [17,18,19], Co-based [20,21,22] and other transition metal catalysts have shown quite amazing performance in the regulation of MgH_2_ hydrogen storage performance. Basile et al. [23] found that doping-free tricyclohexylphosphine (PCy3) and Ni nanoparticles into MgH_2_ can significantly improve the dehydrogenation temperature and kinetic properties of MgH_2_. When only 5 wt% NiPCy3 (0.42 wt% Ni) was doped, the mixture could absorb hydrogen at 220 °C. At the same time, when the hydrogen pressure is 3 MPa and the temperature is 200 °C, the composite can absorb 6.2 wt% H_2_ in 5 min. Gao et al. [24] studied the catalytic performance of two-dimensional Ti_3_C_2_T_x_ MXenes with different crystal faces for the hydrogen absorption and desorption kinetics of MgH_2_. They found that materials with more edge faces exposed had better catalytic activity than materials with basal faces exposed. Strong affinity for hydrogen and high content of in-situ metallic Ti for the edge facets contribute to the enhanced catalytic performance. Chen et al. [25] successfully synthesized Mn nanoparticles and doped them into MgH_2_. Compared to pure MgH_2_, the dehydrogenation temperature of MgH_2_ + 10 wt% Mn composite dropped to 175 °C. Under the hydrogen atmosphere of 3 MPa and 300 °C, the composite can rapidly release 6.7 wt% H_2_ within 5 min. Furthermore, the cycling stability test showed that the composite system has excellent cycling performance, which can still maintain 92% of the initial capacity after 20 cycles. Liu et al. [26] used molecular sieve imidazole framework-67 (ZIF-67) as a precursor to prepare Co nanotubes (Co@CNTs) with uniform size of 10 nm. The novel Co@CNTs can bring the temperature of MgH_2_ down to 267.8 °C. Benefiting from the synergistic effect of Mg_2_Co/Mg_2_CoH_5_ and carbon nanotubes, the composite has good cyclic hydrogen storage performance and fast kinetic performance.

In addition, transition metal oxides have also attracted the attention of researchers due to their good stability in the air environment and easy preparation [27]. Kajiwara et al. [28] used carbon nanotubes (CNTs) as carriers to attach Nb_2_O_5_ to CNTs. The experimental results show that the fibrous CNT on the surface of MgH_2_ matrix may be the reason for the higher cycle stability of MgH_2_-Nb_2_O_5_-CNT composites. At the same time, Nb_2_O_5_ significantly improves the hydrogen absorption and desorption performance of MgH_2_. Bhatnagar et al. [29] prepared Fe_3_O_4_@GS nanoparticles using graphene (GS) as a template and investigated its effect on the hydrogen storage behavior of MgH_2_. MgH_2_:Fe_3_O_4_@GS starts dehydrogenation at about 262 °C and can absorb 6.2 wt% H_2_ within 2.5 min at 290 °C and a hydrogen pressure of about 15.2 bar. Like other oxides, K-based and Mo-based catalysts also showed good performance in improving the kinetic properties of MgH_2_ [30,31,32,33]. M.Ismail et al. [30] prepared MgH_2_ + 5 wt%K_2_SiF_6_ composites by ball-milling. The initial dehydrogenation temperature of the composite system is about 134 °C lower than that of pure MgH_2_. In addition, 5.1 wt% H_2_ can be released within 30 min at 320 °C. The KH, MgF_2_, and Mg_2_Si decomposed from K_2_SiF_6_ during the reaction significantly improved desorption kinetics of the MgH_2_ system as active species. Jia et al. [33] studied the catalytic effect of MoO_2_ and MoS_2_ on the kinetics of hydrogen absorption/desorption of MgH_2_. The results show that both have a positive effect on the desorption temperature of MgH_2_ and can effectively promote the hydrogen absorption and desorption kinetics of MgH_2_. The new species MgS, MgO, and Mo produced in the reaction process play a key role in improving the hydrogen storage performance of MgH_2_. The properties of the above samples are shown in Table 1.

At present, the research on K-based and Mo-based catalysts is not common. Therefore, considering the positive effects of K and Mo on MgH_2_, the preparation of an efficient catalyst of Mo and K bimetallic has become the focus of our attention. The chemical property of K is extremely active, while Mo is a low active element. Thus, the synergism between them is also worth discussing. Different from the traditional hydrothermal synthesis, cubic K_2_MoO_4_ was synthesized by evaporation crystallization method and solid–liquid separation method in this paper. The effect of MgH_2_ on its desorption performance was studied by a series of microscopic characterization methods. Moreover, we also put forward a detailed discussion on the catalytic mechanism. It is believed that this study can provide a strategy for designing efficient hydrogen storage materials and catalysts by using the synergistic effect of metal elements.

## 2. Experimental

The chemicals were purchased from Sinopharm Chemical Reagent Co., Ltd., and can be used directly as is.

### 2.1. Preparation of Cubic K_2_MoO_4_ Co-Reactant

The cubic K_2_MoO_4_ co-reactant was synthesized by a simple evaporative crystallization method and solid–liquid separation method. First, 75.8 g of KOH (AR, 85 wt%) was added to 500 mL ultra-pure water and stirred at high speed for 30 min to form a potassium hydroxide solution. Next, 97.1 g MoO_2_ (trace metals basis, 99 wt%) was added to the mixed solution, and stirring was continued for 30 min to form a potassium molybdate solution. Subsequently, the potassium molybdate solution was evaporated with high-speed stirring at 100 °C until the solution volume was approximately 350 mL. After cooling to room temperature, it was washed with ultra-pure water and absolute ethanol several times to remove unreacted impurities. The amount of deionized water and ethanol used for cleaning are 150 mL and 100 mL, respectively. The resulting product was finally dried at 85 °C overnight. The dried material was taken out and ground repeatedly, and the final product was cubic K_2_MoO_4_ microrods. The prepared co-reactant was stored in the glove box before use to protect the sample from moisture in an argon environment.

### 2.2. Preparation of the MgH_2_ + K_2_MoO_4_ Composite

First, the ambient temperature of hydrogenation of Mg powder is 380 °C when Mg powder was hydrogenated under 65 bar hydrogen pressure for 1 h. Then, Mg powder was put into a ball-milling tank with Ar atmosphere and milled with tungsten carbide balls for 5 h at the speed of 450 rpm. Each large tungsten carbide ball is 7.78 g, each small tungsten carbide ball is 1.68 g, and the ratio of large and small balls is 9:1. Then, the milled powder was crushed and hydrogenated at 380 °C for 2 h [34]. The purpose of crushing is to prevent the agglomeration of powder, which leads to the failure of magnesium powder to fully absorb hydrogen. By repeating the process 5 times, high-purity MgH_2_ powder with hydrogen storage of about 7.2 wt% can be successfully prepared. Subsequently, the prepared K_2_MoO_4_ and MgH_2_ were mixed in proportion into the ball-milling tank and mixed at the speed of 400 rpm for 2 h. The mixed phase must be prepared in Ar atmosphere to prevent MgH_2_ from oxidation and moisture. Finally, three composites with mass ratios of 5:95, 10:90, and 15:85 were successfully prepared, which were recorded as MgH_2_ + 5 wt% K_2_MoO_4_, MgH_2_ + 10 wt% K_2_MoO_4_ and MgH_2_ + 15 wt% K_2_MoO_4_. In order to facilitate the writing of the paper, MK5, MK10, and MK15 are used to represent the three kinds of composites.

### 2.3. Sample Characterization

**Figure 1:**
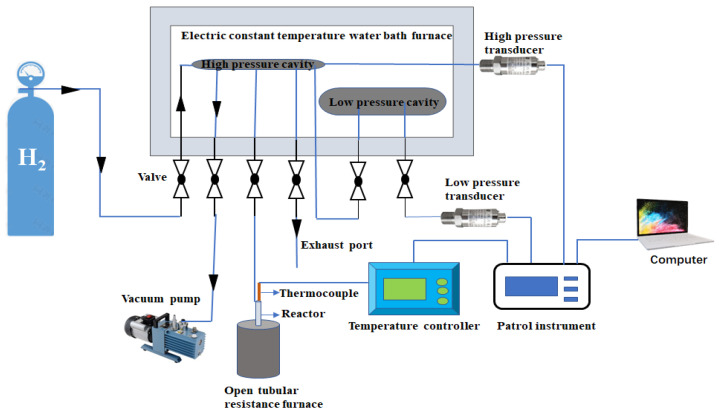
Schematic diagram of hydrogen storage device.

The X-ray direction (XRD) test used a D8 X-ray diffractometer (Bruker). Cu Kα1 and Cu Kα2 were used, the scan rate was 5°/min, and the analysis angle range was 10°–65°. The Scanning electron microscopy (SEM) test used a Hitachi SU-70 device with a working voltage of 30kV. The samples were characterized by transmission electron microscopy (TEM). The type of instrument is FEI TALOS 200X and the working voltage is 200 kV. Jeol JSM-6300 equipment was used for the EDS test. The Differential Scanning Calorimeter (DSC) test used the SAT 449F3 instrument, the test temperature range was 25–450 °C, and the test used Ar as an environmental protection gas. The ab/desorption and cycling tests of the composites were performed using a self-made Sievert-type apparatus with approximately 120 mg of sample per test. The Sievert-type apparatus was mainly composed of integral carbon structural steel (Q235), an electric constant-temperature water bath furnace, and a tubular resistance furnace. The length, width, and height of the apparatus are 88 cms, 46 cms, and 100 cms, respectively. A reactor was installed in the tubular resistance furnace, and the reactor was used to place the samples to be tested. The vacuum pump was used to empty the gas in the low-pressure cavity (850 mL), high-pressure cavity (165 mL), and pipeline during the experiment. The temperature controller can control different heating rates and maintain a constant temperature. The patrol instrument is used to display the pressure value of the sensor and the temperature value of the thermocouple during the test. The high-pressure sensor can withstand up to 20 MPa pressure, and the low-pressure sensor can withstand up to 0.5 MPa pressure. For the accuracy of the test, the high-pressure cavity and low-pressure cavity will always be placed in an electric constant temperature water bath at 25 °C. The detailed schematic diagram of the device is shown in Figure 1. In the dehydrogenation experiment, the temperature of the instrument was increased to 450 °C at a rate of 2 °C/min and kept stable for 10 min. The temperature-rising hydrogen-absorption experiment is to let the instrument heat up to 400 °C at a rate of 1 °C/min and keep it for 10 min under a hydrogen pressure of 3 MPa. The cycle test experiment is to perform repeated hydrogen absorption and desorption operations on the composite material at a constant temperature of 320 °C and a hydrogen pressure of 3 MPa.

## 3. Results and Discussion

### 3.1. Characterization of the Cubic K_2_MoO_4_ Co-Reactant

**Figure 2:**
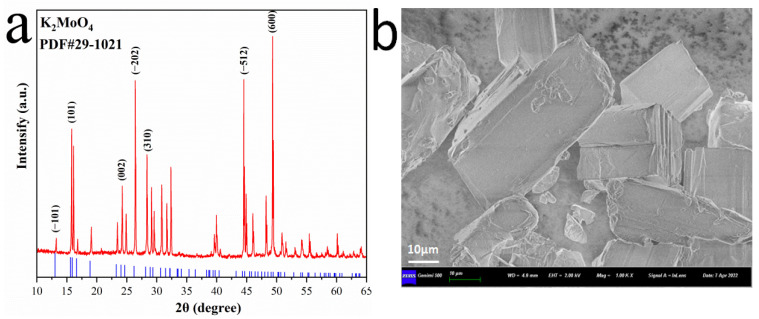

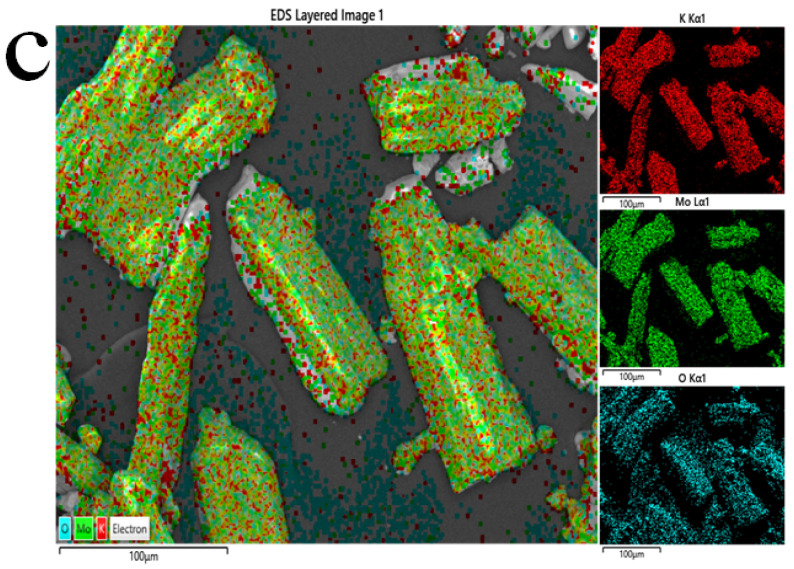
XRD (**a**), SEM (**b**), EDS (**c**) images of cubic K_2_MoO_4_.

The XRD of the cubic K_2_MoO_4_ co-reactant is shown in Figure 2a. The diffraction peaks 2θ of K_2_MoO_4_ mainly appear at 15.6°, 26.2°, 45.8°, and 49.1°, corresponding to its (101), (−202), (−512), and (600) lattice planes, which are consistent with the K_2_MoO_4_ standard card (PDF#29-1021). According to the PDF standard card, the prepared K_2_MoO_4_ belongs to a monoclinic system and the space group is I2/m (12). The number of atoms in the unit cell is Z = 4, and the lattice parameters a, b, and c are 11.340 Å, 6.081 Å, and 7.539 Å, respectively. The special morphology of the co-reactant can affect the exposure of active sites and the resistance of mass transfer, which has a great influence on the catalytic activity. It can be seen from the SEM image in Figure 2b that the K_2_MoO_4_ prepared in this experiment presents a rod-shaped structure, similar to a cube. Moreover, there are defects and discontinuous folds on the co-reactant surface, which is conducive to the exposure of hydrogen evolution active sites. In order to further verify the authenticity and effectiveness of the prepared co-reactant, EDS mapping and elemental analysis were carried out on K_2_MoO_4_. As shown in Figure 2c, it can be clearly seen that the bright spots of different colors in the picture represent K, Mo, and O elements respectively, and no other elements are born. The proportion of K, Mo, and O is 24.3 At%, 12.6 At%, and 63.1 At%, respectively.

### 3.2. Test of Hydrogen Evolution Performance of MK10 Composite System

The dehydrogenation effect of cubic K_2_MoO_4_ on MgH_2_ was verified by isothermal and non-isothermal methods, respectively. The calculation formulas of hydrogen absorption and desorption are shown in Equations (1) and (2), respectively:

Dehydrogenation:

(1) $$\frac{M_{H_{2}}*g*V_{1}*P_{1}}{m*R*T}\;$$

Rehydrogenation:

(2) $$\frac{M_{H_{2}}*g*V_{2}*P_{2}}{m*R*T}\;$$

In the formula, $M_{H_{2}}$ represents the molar mass of hydrogen atom, g represents gravity acceleration, $V_{1}$ represents the volume of high-pressure cavity, $V_{2}$ represents the volume of low-pressure cavity, $P_{1}$ represents the pressure difference of high-pressure cavity, $P_{2}$ represents the pressure difference of low-pressure cavity, m represents sample mass, R represents gas constant, and  T represents the temperature of water bath.

**Figure 3:**
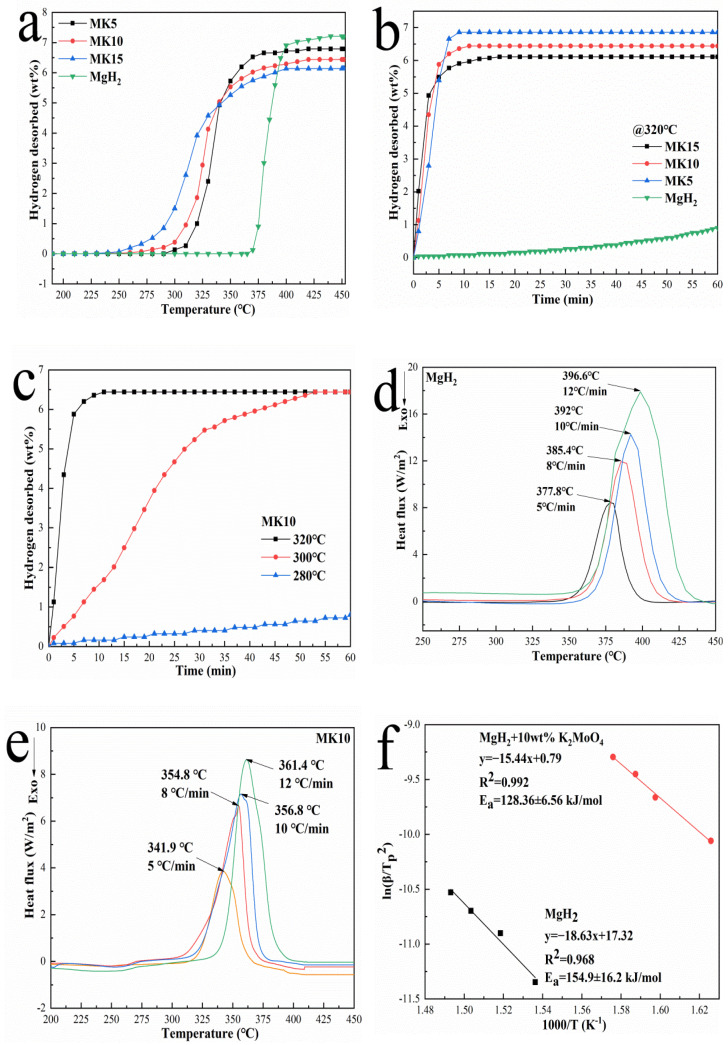
The temperature−rising hydrogen desorption experiment curve (**a**) and isothermal dehydrogenation curve prepared at 320 °C (**b**) for MgH_2_, MK5, MK10 and MK15. Isothermal dehydrogenation curves of MK10 at different temperatures (**c**). DSC curves of MK10 and MgH_2_ (**d**,**e**). Kissinger plots of MgH_2_ and MK10 (**f**).

The main test objects are MK10 composite system and pure MgH_2_. The non-isothermal dehydrogenation curves and isothermal dehydrogenation curves under different co-reactant doping rates are shown in Figure 3a,b. It can be found from the figure that the hydrogen desorption kinetics of MgH_2_ can be significantly improved by doping different proportions of K_2_MoO_4_. The most important thing is that all the composites have achieved complete dehydrogenation. Pure MgH_2_ begins to dehydrogenate at around 360 °C, and the total dehydrogenation capacity at 450 °C is 7.2 wt%. In contrast, the initial dehydrogenation temperature of the composite system is about 250 °C, which is about 110 °C lower than that of pure MgH_2_. It is worth noting that all composite systems can quickly complete all dehydrogenation within 15 min at 320 °C. However, pure MgH_2_ had just started to release hydrogen, and the amount of hydrogen released was about 0.07 wt% under the same condition. Compared to some previous oxide catalysts, K_2_MoO_4_ also showed great progress. Taking the MK10 composite system as an example, MK10 can rapidly dehydrogenate 6.44 wt% at 320 °C within 10 min, reaching the full dehydrogenation amount. In contrast, the dehydrogenation of the composite system doped with 10 wt% LaFeO_3_ is only 3.7 wt% within 15 min at 320 °C [35]. MgH_2_ + 7 wt% NiFe_2_O_4_ can only dehydrogenate 4 wt% in 30 min at 350 °C [36]. For comparison, the effect of using another catalyst on the isothermal desorption kinetics of MgH_2_ is also included in Table 2.

From the perspective of hydrogen storage capacity and reaction rate, the less specific gravity of the co-reactant in the composite material, the higher the hydrogen storage capacity, but the corresponding hydrogen storage rate will also decrease. From Figure 3b, it can be found that the dehydrogenation rate of MK15 is significantly faster than that of the other two composites, and the final dehydrogenation amount of MK5 is the highest. Considering the two factors, MK10 composite material is the most suitable for further study.

Figure 3c shows the isothermal dehydrogenation experiments of MK10 at different temperatures. With the increase in temperature, the dehydrogenation kinetics of the composite system was further improved, and the higher the temperature, the faster the dehydrogenation rate. When the dehydrogenation temperature is 320 °C and 300 °C, MK10 can complete the rapid dehydrogenation within 1 h. However, when the temperature is 280 °C, the dehydrogenation of the composite is only 0.8 wt%. This is mainly because the temperature at this time is close to the initial dehydrogenation temperature (250 °C).

Figure 3d,e show the DSC curves of MK10 and pure MgH_2_ at heating rates of 12 °C/min, 10 °C/min, 8 °C/min, and 5 °C/min. Obviously, the endothermic peak value of doped K_2_MoO_4_ is lower than that of pure MgH_2_ at the same heating rate. The decrease in peak temperature in DSC results showed that the addition of K_2_MoO_4_ effectively improved the analytical performance of MgH_2_. When the heating rate is 5 °C/min, the peak dehydrogenation temperature of MK10 is 341.9 °C, 35.9 °C lower than that of pure MgH_2_.

In order to measure the kinetic performance of dehydrogenation reaction more directly, the apparent activation energy $E_{a}$ of hydrogen evolution reaction of magnesium hydride and its composite system was calculated by the Kissinger method. The calculation formula (Equation (3)) is as follows [48]:

(3) $$\;\;\;\;\;\;\;\;\;\;\;\;\;\;\;\;\;\;\;\;\;\;\;\;\;\;ln(\frac{\beta }{T_{p}^{2}})=-\frac{E_{a}}{RT_{P}}+ln\left(\frac{AR}{E_{a}}\right)\;$$

In the formula, *T_P_* represents the peak temperature in the DSC curve (K), *R* represents the gas constant (J/(kg·K)), *β* represents the heating rate (K/s), and *A* represents the pre-exponential value (s^−1^). *K* is used as the unit in the formula, but in order to facilitate the calculation, °C is used as the unit in the actual experiment.

The Kissinger diagram is shown in Figure 3f. As can be seen from the figure, the slope of curves fitted by MK10 and pure MgH_2_ are 15.44 and 18.63, respectively. For pure MgH_2_ and MK10 composite systems, the apparent activation energy for dehydrogenation are 154.9 ± 16.2 kJ/mol and 128.36 ± 6.56 kJ/mol, respectively. The error is obtained by using the least square method to make the difference between the points of the fitting curve and the actual curve and to find their sum of squares. It is worth noting that the dehydrogenation activation energy of MK10 is about 19% lower than that of pure MgH_2_. Obviously, the addition of K_2_MoO_4_ greatly improves the dehydrogenation kinetics of MgH_2_.

### 3.3. Hydrogen Absorption Performances of MK10 Composite System

The hydrogen absorption behavior of MgH_2_ is another important indicator related to its hydrogen storage performance. The hydrogen absorption kinetics of the MK10 sample after complete dehydrogenation were tested by isothermal and non-isothermal methods under the hydrogen pressure of 3 MPa.

**Figure 4:**
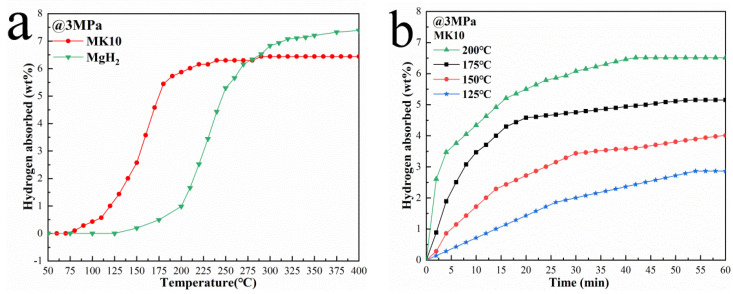

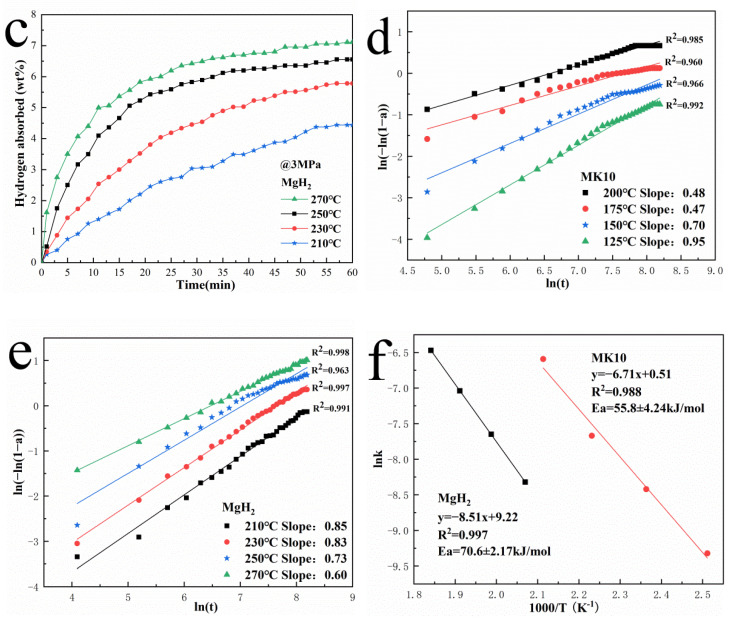
Non−isothermal hydrogen absorption curve of MgH_2_ and MK10 (**a**). Isothermal hydrogenation curves at different temperatures of MK10 (**b**) and MgH_2_ (**c**). JMAK diagrams for MK10 (**d**) and MgH_2_ (**e**). Arrhenius diagrams for MgH_2_ and MK10 (**f**).

Figure 4a shows the curves of hydrogen absorption of pure MgH_2_ and MK10 with temperature. It can be found that the MK10 composite can start to absorb hydrogen below 70 °C. At 200 °C and a pressure of 3 MPa, the hydrogen absorption capacity reaches 5.87 wt%. Finally, it can completely absorb hydrogen at about 290 °C, and the hydrogen absorption capacity is about 6.44 wt%. In contrast, the hydrogen absorption performance of pure MgH_2_ is inferior to that of the doped co-reactant composite system. After complete dehydrogenation, pure MgH_2_ begins to absorb hydrogen at 180 °C, which is about 110 °C higher than that of MK10 composite system. Figure 4b shows the isothermal hydrogen absorption curve of MK10 at different temperatures. Obviously, with the increase in temperature, the hydrogen absorption kinetics of the composite system has been greatly improved. The fully dehydrogenated MK10 composite can absorb 6.46 wt% H_2_ within 40 min at 200 °C. Moreover, nearly 3 wt% H_2_ can be absorbed even at 125 °C. On the contrary, the hydrogen absorption kinetic performance of pure MgH_2_ is much worse. As can be seen from Figure 4c, pure MgH_2_ absorbs only 4.44 wt% H_2_ within 1 h at 210 °C, which is only similar to the hydrogen absorption performance of MK10 composite at 150 °C.

In order to better compare the performance between the two hydrogen absorption systems, the Johnson–Mehl–Avrami–Kolmogorov (JMAK) equation is used to calculate the apparent activation energy ($E_{a}$) of hydrogen absorption required for MgH_2_ to change from a normal state to an active state, which is prone to the chemical reaction. The JMAK equation (Equation (4)) is as follows [49]:

(4) ln(−ln(1−α))=n(ln(k)+ln(t))

where α (wt%) represents the mass fraction of Mg converted to MgH_2_ in time *t* (s), *k* represents the effective kinetic parameter (s^−1^), and *n* represents the Arrhenius Avrami index.

Figure 4d,e show the JMAK curves of MK10 composite and pure MgH_2_, respectively. The functional relationship between ln(−ln(1−α)) and ln(t) is based on the isothermal hydrogen absorption data at different temperatures. It can be found that all curves have a good fitting, and the value of R^2^ is more than 0.96, which has high reliability. In this case, it is easy to calculate the slope *n* of the fitting line at different temperatures.

Subsequently, the apparent activation energy of the hydrogen absorption process is calculated by the Arrhenius equation. The functional relationship between 1000/T and lnk is drawn and fitted again. Finally, the apparent activation energy ($E_{a}$) of the hydrogenation reaction is calculated from the linear slope. The Arrhenius-specific equation (Equation (5)) is as follows [50]:

(5) $$k=Aexp\left(-\frac{E_{a}}{RT}\right)$$

As shown in Figure 4f, the fit of all the curves is good, and the slopes of the fitted curves for pure MgH_2_ and MK10 are 8.51 and 6.71, respectively. It can be calculated that the activation energy of hydrogen absorption of MgH_2_ is 70.6 kJ/mol, and the activation energy of hydrogen absorption of MK10 is 55.8 kJ/mol. Compared to pure MgH_2_, the activation energy of the composite system decreased by more than 20%. The decrease in activation energy shows that K_2_MoO_4_ as a co-reactant does reduce the chemical energy barrier of MgH_2_ in the hydrogen absorption process. This is also the main reason for the significant improvement of MgH_2_ hydrogen absorption kinetics.

### 3.4. Cyclic Stability Test of MK10 System

**Figure 5:**
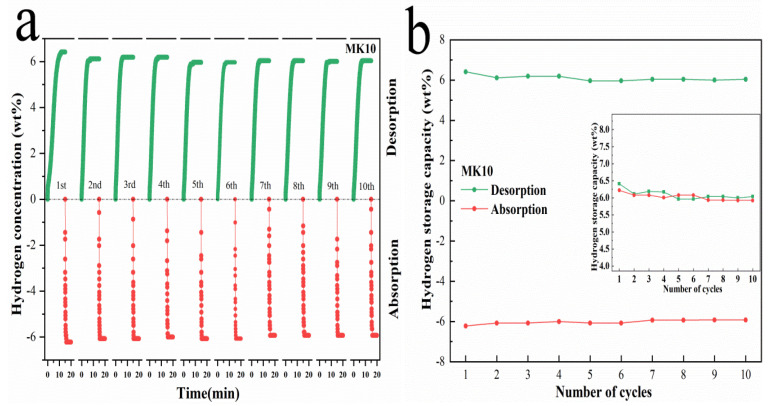

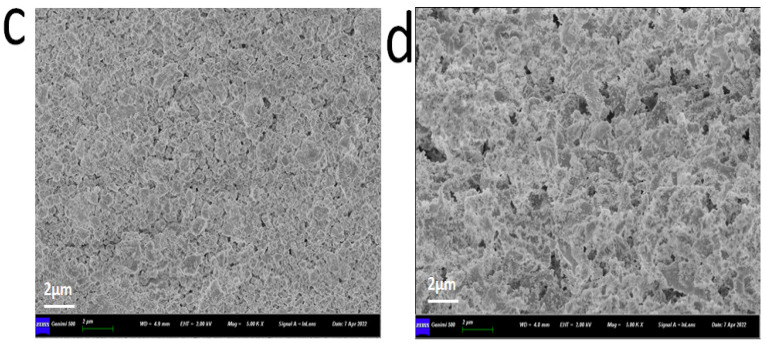
Hydrogen ab/desorption cycle curves of MK10 at 320 °C (**a**). Curves of hydrogen storage capacity and hydrogen release capacity of composites for 10 cycles (**b**). The SEM diagrams of MK10 after ball-milling (**c**) and the10th cycle (**d**).

Cyclic stability is a significant parameter for evaluating the performance of hydrogen storage, and it is also one of the important indicators for the practical application of MgH_2_. In order to study the cycling stability of MK10 composites, 10 cycles of absorption and dehydrogenation were carried out at a constant temperature of 320 °C, and the cyclic hydrogen pressure is 3MPa. As shown in Figure 5a, the hydrogen desorption time for each cycle is 15 min and the hydrogen absorption time is 5 min. During the first dehydrogenation, the hydrogen desorption amount is 6.41 wt%, and the hydrogenation amount is 6.22 wt%. The second cycle decay is more serious, the amount of hydrogen desorption is reduced to 6.11 wt%, and the amount of hydrogen absorption is reduced to 6.08 wt%. As the number of cycles increases, the attenuation begins to decrease. From the 5th cycle, the amount of hydrogen released and absorbed tends to be flat until the 10th cycle is basically stable. When the 10th cycle is completed, the hydrogen absorption and desorption are 6.04 wt% and 5.92 wt% respectively, and the composite can still maintain about 92.3% hydrogen storage capacity.

It can be seen from Figure 5b that the composite material showed a relatively obvious decline in the second time, and the hydrogen storage capacity decreased to about 95.3% of the original. This means that in the following eight cycles, the hydrogen storage capacity declined by only 3%. In addition, it can be found that the hydrogen storage capacity during the cycle is not declining all the time but fluctuates up and down. It may be related to experimental error in the measurements during long runs. Similar phenomena have also been reported in other literatures [51]. In general, the cyclic properties of MK10 composites are relatively stable.

Further analysis shows that the decline and fluctuation of the cycle process may be caused by the agglomeration and growth of composites in a high temperature environment [52]. It can be seen from the SEM images in Figure 5c,d that the MK10 composite before cycling is finely and evenly distributed. After 10 cycles, the composites showed a significant increase in size and agglomeration. The agglomeration problem will lead to the abnormal growth of particles, and may change the structure, resulting in the deterioration of material properties.

### 3.5. Catalytic Mechanism of K_2_MoO_4_ in Hydrogen Absorption and Desorption

**Figure 6:**
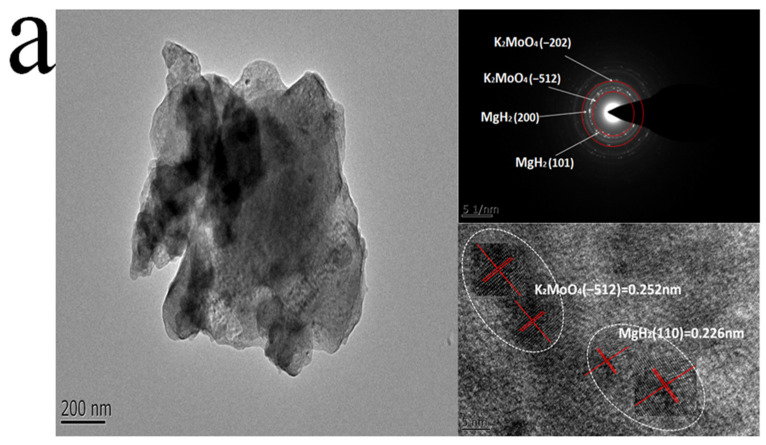

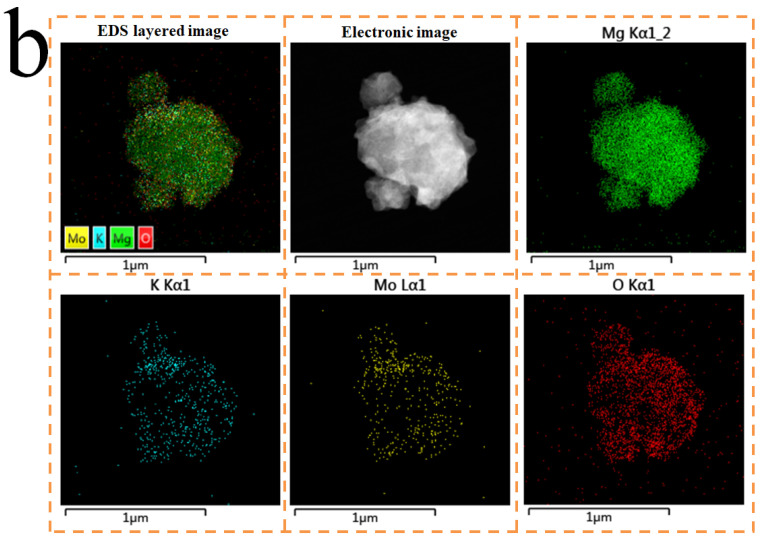
A TEM diagram with a HRTEM pattern and SAED image of MK10 after ball-milling (**a**). The EDS diagram of MK10 after ball-milling (**b**).

The actual catalytic efficiency of K_2_MoO_4_ for MgH_2_ is usually related to the distribution and activity of the co-reactant. The microstructure of the ball-milled MK10 was further studied by TEM, HRTEM, SAED, and EDS. The lattice spacings of the MgH_2_ (110) and K_2_MoO_4_ (−512) surfaces were shown in the HRTEM diagram in Figure 6a, and the calculated distances were 0.226 nm and 0.252 nm, respectively, which were in good agreement with the XRD data. In addition, the diffraction rings of K_2_MoO_4_ (−512), K_2_MoO_4_ (−202), MgH_2_ (101), and MgH_2_ (200) can also be clearly observed in the SAED diagrams. The EDS test in Figure 6b further proves the stability of the K_2_MoO_4_ co-reactant during ball-milling. The results show that the composite only contains Mg, K, Mo, and O, and no other new elements are discovered. At the same time, K_2_MoO_4_ basically covers the entire composite material after the material is ball-milled, which is beneficial to the formation of an active system for releasing hydrogen and the increase in catalytic sites.

**Figure 7:**
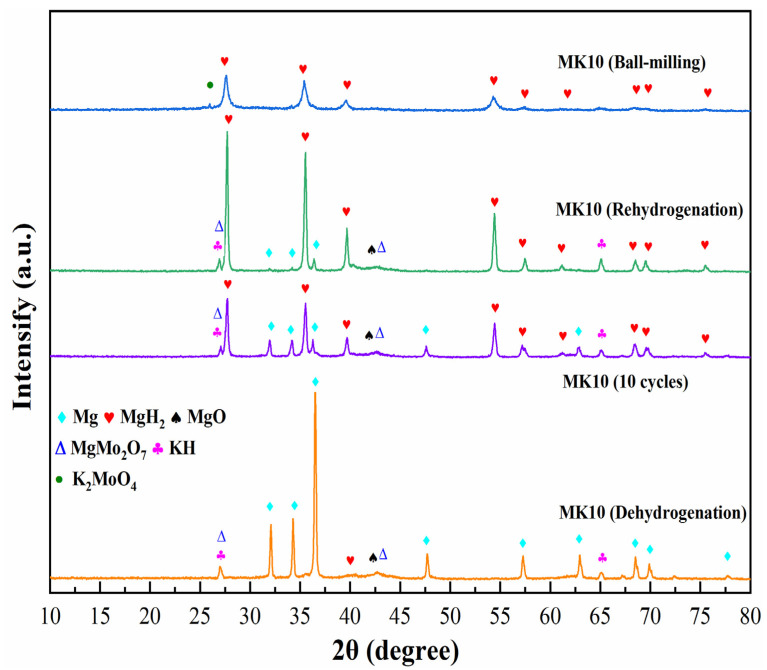
XRD patterns of MK10 in four different stages: ball-milled state, dehydrogenated state, hydrogenated state, and the stage after 10 cycles.

Although the above studies have found that K_2_MoO_4_ can improve the de/rehydrogenation performance of MgH_2_, the mechanism of improving its kinetic performance needs to be further explored. Therefore, the samples of MK10 were characterized by XRD at all stages, including hydrogen absorption, hydrogen desorption, and 10 cycles. The results were compared to the newly prepared samples. It can be seen from Figure 7 that the main component of the composite material after ball-milling is MgH_2_. The dark green dots appearing at 26.2° is K_2_MoO_4_ co-reactant, which is consistent with the XRD patterns of K_2_MoO_4_ prepared in Figure 2a. No new phase appeared in the composite except MgH_2_, Mg, K_2_MoO_4_, which indicated that the co-reactant was stable and no reaction took place after ball-milling. When the MK10 composite is completely dehydrogenated, the main diffraction peaks are changed to Mg, which indicates that the dehydrogenation process is relatively smooth. 

**Figure 8:**
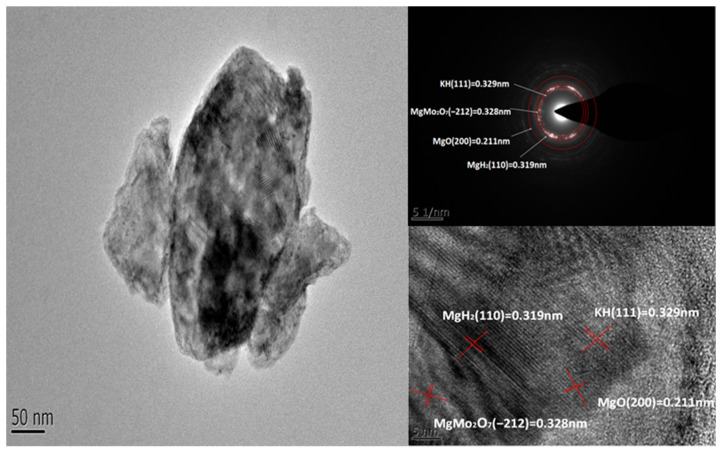
A TEM diagram with a HRTEM pattern and SAED image of MK10 after rehydrogenation.

Interestingly, the peak of the co-reactant K_2_MoO_4_ disappeared, and three new species of KH, MgO, and MgMo_2_O_7_ appeared, which indicated that MgH_2_ reacted with K_2_MoO_4_. In order to further verify the existence of new species, we characterized the samples that completed a complete hydrogen absorption and desorption cycle, as shown in Figure 8. The strongest intensity peaks of each materials are KH (111) (*d* = 0.329 nm, Intensity 99, 2θ = 26.9°), MgMo_2_O_7_ (−212) (*d* = 0.328 nm, Intensity 97, 2θ = 27.1°), MgH_2_ (110) (*d* = 0.319 nm, Intensity 100, 2θ = 27.9°), and MgO (200) (*d* = 0.211 nm, Intensity 99, 2θ = 42.9°). Their chemical reaction equations are shown in Equations (6) and (7):

(6) $$2K_{2}{\mathrm{MoO}}_{4}+2{\mathrm{MgH}}_{2}\to \mathrm{MgO}+4\mathrm{KH}+\mathrm{Mg}{\mathrm{Mo}}_{2}O_{7}$$

(7) $${\mathrm{MgH}}_{2}⇋\mathrm{Mg}+H_{2}↑$$

When the composite materials absorb hydrogen again, the main diffraction peak became MgH_2_, which means that the reaction process is reversible—one of the key features necessary for hydrogen storage. In addition to MgH_2_, there are also many diffraction peaks of Mg, which may be due to the agglomeration and growth of the material. Reactions in high temperature environments tend to cause material sintering, resulting in larger crystallite size. This is also the main reason why part of Mg is not completely hydrogenated to MgH_2_. The XRD diffractogram of the composite after cycling further confirms this conclusion. It can be clearly found that the diffraction peak of Mg becomes more obvious and the number increases after 10 cycles. When part of Mg cannot be fully hydrogenated to MgH_2_ after hydrogenation, the hydrogen storage capacity of the composite system will decrease, which is consistent with the results of previous cycle-stability tests.

In addition, the in situ formation of KH, MgO, and MgMo_2_O_7_ during the desorption process may have additional catalytic effects on the hydrogen storage performance of MgH_2_, which can provide a shorter hydrogen path for faster hydrogen absorption and desorption processes. They remain unchanged in repeated hydrogen absorption and desorption experiments, and play a real catalytic role. Notably, KH as an active species may have played a key role in the hydrogen storage performance of MgH_2_. The reason for this conjecture is that KH positively affects both Mg(NH_2_)_2_/2LiH and 2LiNH_2_–MgH_2_ systems. Due to the addition of KH, the decomposition temperature of the Mg(NH_2_)_2_/2LiH system can be reduced by about 50 °C. Furthermore, the Li_2_Mg(NH)_2_ system catalyzed by KH can be fully hydrogenated within 6 min, while pure Li_2_Mg(NH)_2_ can only hydrogenate 70% within 30 min [53,54]. 

## 4. Conclusions

This paper mainly studies the improvement of hydrogen storage performance of the MgH_2_ system by K_2_MoO_4_ oxide. Specifically, MK10 begins to dehydrogenate at around 250 °C, which is about 110 °C lower than that of the newly prepared MgH_2_. During the isothermal dehydrogenation experiment, the MK10 composite can be completely dehydrogenated within 10 min at 320 °C, showing extremely fast dehydrogenation kinetics. In contrast, pure MgH_2_ releases only a little hydrogen under the same conditions. Moreover, the fully dehydrogenated composite can absorb hydrogen again at about 70 °C, while the pure MgH_2_ is 110 °C higher. At 200 °C and a hydrogen pressure of 3MPa, the MK10 composite can rapidly absorb 5.5 wt% H_2_ within 20 min. On the other hand, compared to pure MgH_2_, the activation energy of hydrogen absorption and hydrogen desorption of MK10 composites decreased by 14.8 kJ/mol and 26.54 kJ/mol, respectively. The cycle performance test results show that the composite material has good stability. No significant decrease was found after five cycles, and the hydrogen storage capacity of the composite system was still above 92% after ten cycles. Catalytic mechanism studies show that K_2_MoO_4_ is uniformly covered on the surface of MgH_2_ substrate. Three new substances, KH, MgO, and MgMo_2_O_7_, were formed during the reaction, which synergistically improved the hydrogen storage properties of MgH_2_. They provide shorter hydrogen paths for faster hydrogenation processes and increase more catalytically active sites, thereby reducing the energy barriers for H dissociation and absorption. It is believed that this work can enrich the research on existing hydrogen storage systems to a certain extent and provide new ideas for the development and design of high-performance hydrogen storage materials.

## Figures and Tables

**Table 1:** Hydrogen absorption and desorption properties of different composites.

Sample	Hydrogen Absorption	Capacity (wt%)	Hydrogen Desorption	Capacity(wt%)	T_onset_ (°C)	Refs.
MgH_2_ + 5 wt%NiPCy3 (0.42 wt%Ni)	30 bar + 200 °C + 5 min	6.2	-	-	220	[23]
MgH_2_−5 wt% F-Ti_3_C_2_T_x_	3 MPa + 125 °C + 1200 s	4.57	275 °C + 1000 s	5.95	199	[24]
MgH_2_ + 10 wt% Mn	3 MPa + 100 °C + 30 min	3.3	275 °C + 10 min	6.5	175	[25]
MgH_2_ + Co@CNTs	30 bar + 250 °C +2 min	6.15	280 °C + 30 min	6.15	267.8	[26]
MgH_2_-Nb_2_O_5_-CNT/SUS MgH_2_: Fe_3_O_4_@GS	1.9 Mpa + 350 °C + 100 s 15 atm + 290 °C + 2.5 min	5.91 6.2	350 °C + 100 s 290 °C + 20 min	5.81 6.0	−262	[28] [29]
MgH_2_ + 5 wt% K_2_SiF_6_	30 atm + 250 °C + 2 min	4.5	350 °C + 60 min	5.4	282	[30]
MgH_2_ + 16.7 wt% MoO_2_	3 MPa + 423 K + 31 min	2.45	573 K + 10 min	0.23	376.9	[33]
MgH_2_ + 16.7 wt% MoS_2_	3 MPa + 423 K + 13 min	3.01	573 K + 10 min	0.57	367.2	[33]

**Table 2:** Isothermal desorption kinetics of different composite systems.

Sample	Onset Temperature (°C)	Conditions	Capacity (wt%)	Refs.
MgH_2_ + 10 wt% LaFeO_3_	300	320 °C + 15 min	3.7	[35]
MgH_2_ + 10 wt% MgNiO_2_	258	320 °C + 10 min	5.1	[37]
MgH_2_ − 10 wt% SrTiO_3_/5 wt%Ni	260	320 °C + 13.1 min	6.6	[38]
MgH_2_ + 10 wt% Co_2_NiO	300	320 °C + 60 min	4.0	[39]
MgH_2_ + 10 wt% SrFe_12_O_19_	270	320 °C + 15 min	4.3	[40]
MgH_2_ + 16.7 wt% BiVO_4_	265	300 °C + 20 min	1.1	[41]
MgH_2_ − 10 wt% SrTiO_3_	275	320 °C + 12.3 min	4.6	[42]
MgH_2_ + 10 wt% BaFe_12_O_19_	270	320 °C + 15 min	3.5	[43]
MgH_2_ + 4 mol% ZrO_2_	260	400 °C + 20 atm	~5	[44]
MgH_2_ + 4 mol% CeO_2_	270	400 °C + 20 atm	~5	[44]
MgH_2_ + 5 mol% CuO	−	300 °C + 25 min	6	[45]
MgH_2_ + 1 mol% BaRuO_3_	−	320 °C + 30 min	4.6	[46]
MgH_2_ + 5 wt% NiO/Al_2_O_3_	240	300 °C + 60 min	~6	[47]
MK10	250	320 °C + 10 min	6.44	This work

## Data Availability

Not applicable.
